# Genome-Wide Search Reveals the Existence of a Limited Number of Thyroid Hormone Receptor Alpha Target Genes in Cerebellar Neurons

**DOI:** 10.1371/journal.pone.0030703

**Published:** 2012-05-07

**Authors:** Fabrice Chatonnet, Romain Guyot, Frédéric Picou, Maria Bondesson, Frederic Flamant

**Affiliations:** 1 Institut de Génomique Fonctionnelle de Lyon, Université de Lyon, Université Lyon 1, CNRS, INRA, École Normale Supérieure de Lyon, Lyon, France; 2 Karolinska Institute Novum, Stockholm, Sweden; Laboratoire Arago, France

## Abstract

Thyroid hormone (T3) has a major influence on cerebellum post-natal development. The major phenotypic landmark of exposure to low levels of T3 during development (hypothyroidism) in the cerebellum is the retarded inward migration of the most numerous cell type, granular neurons. In order to identify the direct genetic regulation exerted by T3 on cerebellar neurons and their precursors, we used microarray RNA hybridization to perform a time course analysis of T3 induced gene expression in primary cultures of cerebellar neuronal cell. These experiments suggest that we identified a small set of genes which are directly regulated, both *in vivo* and *in vitro*, during cerebellum post-natal development. These modest changes suggest that T3 does not acts directly on granular neurons and mainly indirectly influences the cellular interactions taking place during development.

## Introduction

3,5,3′ tri-iodothyronine (T3), the active form of thyroid hormone, acts by binding to its cognate receptors, thyroid hormone receptors (TRs), which are transcription factors from the nuclear receptor family [1]. TRs exist as three isoforms, TRα1, TRβ1 and TRβ2, encoded by the *Thra* and *Thrb* genes respectively. They can bind DNA both in the presence and absence of T3, either as homodimers or as heterodimers with RXR (Retinoid X Receptor). DNA binding occurs on so-called T3 response elements (TREs) which usually associate two consensus half-sites (5′AGGTCA3′) often organized either as direct repeats separated by four nucleotides (DR4). T3-liganded TRs have the ability to induce either transcriptional activation or repression depending on the locus and the local environment. For positive regulation, unliganded TRs change conformation upon T3 binding, releasing transcription corepressors to recruit coactivators and leaving epigenetic marks on neighboring histone tails [2]. Negative regulation mechanisms are poorly understood, and still a matter of controversy.

The cerebellum is a very suitable structure to study T3 action during neurodevelopment because it is particularly sensitive to hypothyroidism [3]. Moreover, the cerebellum has a relatively simple architecture compared to other brain regions, with only a limited number of cell types. The cerebellar cortex is organized in layers lying over the white matter and the deep cerebellar nuclei. The cerebellar cortical layers comprise of the molecular layer containing GABAergic interneurons, the Purkinje cell layer that also contains the cell bodies of Bergmann glia cells and the internal granular cell layer (IGL), containing mature granular cells, astrocytes and Golgi GABAergic interneurons. During development, granular cell precursors form a germinative layer over the surface of the cerebellar primordium called the external granular layer (EGL), where precursors divide actively and start their differentiation after exiting the cell-cycle (for review see [4]). Post-mitotic granular neurons extend their axon in the molecular layer and migrate their cell body through the Purkinje cell layer to populate the IGL. At post-developmental stages, granular neurons represent more than ninety percent of cerebellar cells.

T3 deprivation during cerebellar post-natal development affects all these cell types (reviewed in [5]). Granular cell migration is blocked and granular cell precursors are trapped in the EGL after the third post-natal week, when migration towards the IGL is normally finished [6]. Purkinje cell layering is defective and the size of their dendritic tree is reduced [7]. Bergmann glia fibers morphology is abnormal, and astrocytes [8], [9] show an over-proliferation and delayed differentiation. Oligodendrocytes [10], [11] and GABAergic interneurons [12] differentiation is delayed. Mouse genetics provided clear evidences that most if not all of these defects are due to the presence of unliganded TRα1 in all cerebellar cell types. First, *Thra* knock-out reverses the deleterious effect of hypothyroidism [13]. Second, dominant negative mutations of TRα1, which affect either its ability to bind T3 or to recruit coactivators, lead to a cerebellar phenotype resembling hypothyroidism [14], [15], [16]. Unlike *Thra, Thrb* is expressed in only a few cell types and at low level in the developing cerebellum. However, *Thrb* point mutation has also been found to induce important cerebellar defects [17], raising the possibility that T3 can initially act on few cell types, and indirectly exert a global and indirect influence on a network of cellular interactions.

Several attempts have been performed to elucidate the molecular events underlying the observed cellular alterations in hypothyroid cerebellum and identify the TRs target genes. Various animal models have been used to identify genes which expression levels are sensitive to T3 status [18]–[22] or which promoters are directly bound by TR [23]. Although a number of variations in mRNA levels were observed, most of these experiments failed to establish a direct link between cells behavior and TR regulated transcription, as changes in gene expression might be explained by variations in neurotrophic factors levels, which are secondary to T3 deficiency [21]. Cellular heterogeneity of the cerebellum, which can mask variations in gene expression restricted to one cell type, is also likely to impair the identification of direct TR target genes in these experiments. Primary neuronal cell cultures in part circumvent these problems as they allow focusing on early events triggered by T3, limiting cellular interactions and favoring neurons or glial cells by choosing adequate culture conditions and thus reducing cellular heterogeneity. Up to now, and to our knowledge, RNA analysis of cultured cells has demonstrated the direct upregulation by TRα1 (bound to identified TREs) of only four genes, which are deregulated in hypothyroid cerebellum: *Hairless (Hr)*, *synaptotagmin-related gene 1 (Srg1),* [24] *Krüppel-like factor 9 (Klf9)* [25], [26] and *A kinase anchor protein 1 (Akap1)* [22]. To reach a better understanding of the genetic regulation underlying the neuronal defects observed in hypothyroid brain, we performed a genome-wide transcriptome analysis of T3 response in primary cerebellar neurons culture. This identified 15 genes, which are either up- or down-regulated by TRα1 in response to T3. We further studied whether these genes are directly regulated by TRα1 *in vitro* and *in vivo* in different cell types of the cerebellum and in a cerebellum cell line. We chose to work on the C17.2 cell line [27] since it is derived from EGL cells and thus was supposed to be closely related to our primary cultures. We used this cell line to have a more convenient access to chromatin binding since it is easier to realize chromatin precipitation in cells rather than in tissues, particularly in our case where there is no “ChIP grade” antibody for TRα1.

## Results

### Genome-wide Search of T3 Regulated Genes

In order to identify changes in gene expression which are mainly related to immediate and direct action of thyroid hormone on post-natal cerebellum neuronal cells, we prepared primary cultures of cerebellar cells and maintained them in conditions favoring neurons survival, at the expense of glial cell proliferation. It has been shown that in this system, granular neurons, which represent the majority of cerebellar cells, switch from proliferation to migration and finally terminal differentiation, including dendritogenesis [28], thus reproducing the normal stages of development. We verified by immunocytochemistry that in these conditions most of the cells (80%) express the Tuj1 neuronal markers, and that prolonged culture (10 days) allowed the cells to grow fine dendritic-like processes. Two days after seeding, cells were treated with T3 for various amounts of time (6, 16, 24 or 48 hours) or left untreated before RNA extraction. Total RNAs were then amplified and cDNA prepared to serve as probes for microarrays hybridization. (See dataset GSE24793 on the NCBI Gene Expression Omnibus (GEO), http://www.ncbi.nlm.nih.gov/geo/).

Only probe-sets with a sufficient level of expression (P in columns “detection” of data matrix available at the GEO depository, covering about 40% of all annotated genes) and that showed a fold change superior to 2 were considered. We detected signals for markers for each cerebellar cell-type, indicating that our culture system contained all the cell constituents of the cerebellum and we also found that its composition evolved over time. For example, at early time points we detected high levels of *Pax6* and *Ccnd2*, two markers of granule cell precursors, which decreased over time. Conversely, we observed low levels of *Gabra6*, a marker of differentiated granule cells, which increased over time. We also detected *Pcp2* expression, a marker of Purkinje cells, *Pdgfra* and *Plp1*, markers of oligodendrocyte precursors and of mature oligodendrocytes, respectively, and *Slc1a3* (Glast) and *Gfap*, two markers of Bergmann glia and astrocytes.

To address the robustness of T3 regulation in primary cultures of cerebellar cells, we used Q-RT-PCR to compare the microarray results acquired with a first set of primary cultures to RNA expression of both the same and another independent primary cultures experiment. We generated primers for the most up- or down-regulated genes for each time point and for genes showing an overall constant tendency (up- or down-regulated for all time points) from the microarray results. Given the relatively low numbers of probe-sets corresponding to these criteria, we also selected genes showing a change in expression of at least 1.6 fold for one or more time points, and genes that were previously known to be regulated by T3, like *Ccnd2* or *Hr*. We confirmed that T3 induced changes in gene expression for a number of genes and the changes in expression pattern increased over time. 6 genes were up-regulated and 9 were down-regulated in a significant and reproducible manner (Table 1). We focused later investigations on this group of 15 putative TR target genes The group of up-regulated genes included the previously reported T3 target gene *Klf9*. *Hr* expression was too low to be detected by microarray hybridization, but its expected induction by T3 was confirmed by Q-RT-PCR. This result is consistent with Q-RT-PCR being a more sensitive detection method of RNA than microarray hybridization, and indicates that our survey, although revealing expression of about 40% of the annotated genes in the cultured cells, might have missed some changes in gene expression, due to limited sensitivity. However, detection of mRNA expressed by very limited cell populations (see above, ie, *Pcp2* in Purkinje cells) suggests that it was not the case.

**Table 1:** Q-RT-PCR confirmation of T3 mediated regulation in primary cultures of cerebellum neuronal cells.

Gene	*Full name*	Microarrays				Q-RT-PCR		
		6h	16h	24h	48h	6h	16h	24h
*Anxa8*	*Annexin 8*	1,16	0,62	1,41	0,54	0,92	1,12	0,67
***Dbp***	*D-site albumin promoter binding protein*	**2,65**	**3,06**	**2,47**	1,87	**2,14**	1,87	1,75
***Gbp3***	*Guanylate binding protein 3*	1,41	1,62	**3,48**	1,74	1,30	1,85	**2,50**
***Igsf3***	*Immunoglobulin superfamily, member 3*	0,80	0,80	**0,36**	0,90	1,01	0,98	0,76
***Klf9***	*Krüppel-like factor 9*	**2,49**	1,92	**3,09**	1,76	**5,48**	**9,21**	**4,04**
***Pfkfb3***	*6-phosphofructo-2-kinase/fructose-2,6-biphosphatase 3*	**2,00**	1,32	1,30	1,27	1,36	1,93	1,62
***Plp1***	*Proteolipid protein 1*	1,55	1,33	**0,37**	**2,19**	1,13	1,09	0,92
*Plxna2*	*Plexin A2*	1,94	0,67	1,09	1,29	0,97	1,04	0,97
***Snap25***	*Synaptosomal-associated protein, 25 kD*	1,15	1,19	1,08	1,76	1,98	**2,06**	**2,92**
***Spata13***	*Spermatogenesis associated 13*	**2,05**	1,63	**2,06**	1,39	**2,62**	**2,00**	**2,58**
*Tgm2*	*Transglutaminase 2*	0,93	1,05	1,48	0,64	1,09	1,17	0,90
*Vnn1*	*Vanin 1*	0,93	0,52	1,46	0,62	1,13	0,94	N/A
***Zbtb20***	*Zinc finger and BTB domain containing 20*	1,21	1,21	1,25	1,37	1,58	1,26	**2,82**

Bold characters outline fold changes superior to 2.

### In vitro Study of Target Genes Regulation of Expression

**Figure 1:**
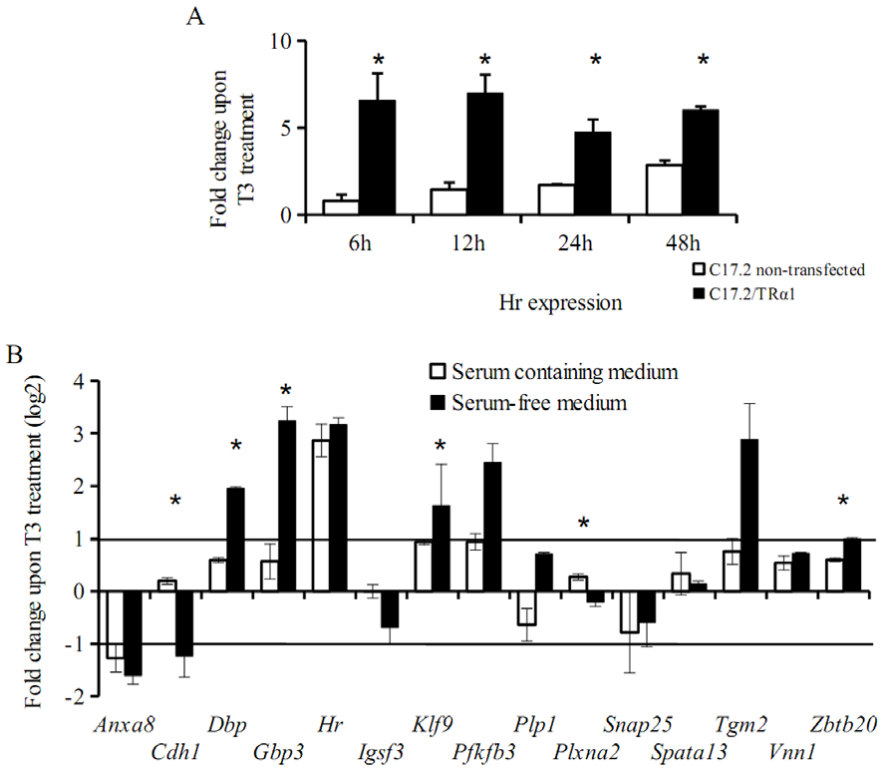
Transfection of TRα1 into C17.2 cells restores their response to T3 treatment. A. T3-induced *Hr* expression is detected earlier and stronger in transfected C17.2/TRα1 cells (black bars) than in non-transfected cells (white bars), *p<0.05, Student’s t-test difference between non-transfected cells and C17.2/TRα1 cells. B. The level of change in expression induced by T3 and measured by Q-RT-PCR in C17.2/TRα1 cells is indicated for each target gene, using non-treated cultures as reference (represented as log2 of the fold change). White bars indicate T3 treatment in proliferative medium (containing serum), black bars indicated T3 treatment in serum-deprived medium allowing for differentiation. Most genes show a response only in serum-deprived medium, *p<0.05, Student’s t-test, difference between serum containing and serum deprived cultures.

The fact that most of the genes that we identified were distinct from those that have been identified in previous studies prompted us to address the possibility that some genes might be regulated in specific cell types, or at specific differentiation steps, whereas others would be regulated more broadly. To study the expression of the T3 regulated genes on another cellular background we used C17.2 cerebellum immortalized pluripotent cell line, which originates from EGL [27]. Although the exact nature of these cells is unclear [29], they are able to differentiate into granular-like neurons when grafted in brain [30]. We first restored C17.2 cells response to T3 by expressing a tagged version of TRα1 in a stable manner and measured the expression of *Hr* in response to TR and T3 (Fig. 1A). We found that both C17.2 and C17.2/TRα1 cells can differentiate within 5 days to Tuj1+ neuronal-like cells in serum-free Neurobasal medium. Interestingly, in this differentiation assay, the presence of T3 completely prevented the appearance of Tuj1+ cells in C17.2/TRα1 but not in C17.2 (data not shown). We then addressed the expression of the 15 novel TR target genes, identified in primary neuronal cultures, in C17.2/TRα1 cells after 48 hours of T3 stimulation in the presence or absence of serum. Figure 1b indicates that, soon after serum deprivation, T3 regulation was visible in these cells, with 3 exceptions (*Plxna2, Spata13* and *Vnn1*). Interestingly, the T3-induced changes in gene expression in presence of serum were mainly limited to *Hr* induction. Although thought to be expressed only in the oligodendrocytes lineage, *Plp1* was activated in serum starved C17.2/TRα1 cells, which did not express other typical oligodendrocyte markers, like *Mbp* gene, encoding the Myelin Basic Protein (data not shown).

The C17.2/TRα1 cell line appears therefore as a convenient *in vitro* model to study the T3/TRα1 signaling pathway in a neural context. It offers the possibility to assess if TRα1 is bound to consensus binding sequences (TR response element, TRE) at proximity of the target genes transcription start site (TSS). Using previous bioinformatics data [22], putative TREs conserved between mouse and human were first identified within 25 kb of transcription start sites (TSS). When no conserved sequence was found, Nubiscan online tool (http://www.nubiscan.unibas.ch/) was also used to extend the search to near-consensus sequences in this region. This gave us a list of putative TREs for each locus (Table 2). Taking advantage of the proteinG tag present on TRα1, we performed Chromatin Affinity Purification (ChAP) to enrich fractionated chromatin into TRα1 bound complexes. This confirmed the presence of TRα1 in areas covering some of previously reported TREs, upstream to *Klf9* [26], *Hr* [22] and *Dbp* [23], and one downstream to the transcription start site of *Gbp3* (Table 2). The transcriptional regulation for these genes is thus likely to result directly from the activation of TRα1 bound to neighboring regulatory sequences. We also used a recombination mediated cassette exchange assay [31] to address the ability of upstream promoter sequences of these genes to drive the stable expression of *renilla luciferase* in C17.2/TRα1 cells and mediate T3 regulation in a physiological chromatin context. This confirmed the presence of T3 regulatory elements within 6 kb of upstream sequences for *Hr* but not for *Gbp3* and *Anxa8* (data not shown).

**Table 2:** Chromatin occupancy by TRα1 in C17.2/TRα1 cells.

Gene	Conserved TRE position	Type TRE	Sequence	TRα1 affinity precipitation (enrichment)
*Anxa8*	−13935	DR4	GTGTCAtgcaAGGTCA	0.74±0.13
	−8825	DR4	GGGGCAtccgAGTTCA	0.69±0.12
	−7406	DR4	AGGTCActccAGGTCC	ND
	−314	DR4	AGTTCAgcaaAGGACA	0.66±0.19
*Cdh1*	−9477	DR4	AGTTTAtgtaAGGTCA	ND
	−2600	DR4	GGGACAgaaaGGGTCA	0.88±0.03
	+1784	DR4	AGGTCAttttGGTGCA	1.48±0.34
	+14093	DR4	GGGTCAccaaGTGTCA	1.15±0.22
	+19817	DR4	AGGTCAcgggAGTTAA	1.12±0.23
	+22206	DR4	AGTTCAagtgAAGTCA	0.98±0.07
	+8445	ER6	TGTCCTcagggaAGTTCA	1.15±0.17
*Dbp*	−17879	DR4	TGGTCAtagcAGGTCA	1.23±0.10
	−10039	DR4	GGGTTAaggaAGTTCA	1.03±0.08
	+18318	DR4	AGGTCActggGGTTCC	1.14±0.15
	**−341**	**DR4/ER6**	**TTGGCCAAatatAGGTCA**	**2.57±0.04**
*Gbp3*	**+406**	**DR4/ER6**	**TCAGCTCAgctgAGGTCA** *	**5.5±0.14**
	+10643	IR0	AGGTCATGACCT*	1.2±0.23
*Hr*	**−2345**	**DR4**	**AGGGCAtctgAGGACA** *	**9.5±1.12**
*Igsf3*	+8259	DR4	AAGTCAactgAGGTCA	1.77±0.22
	+9904	DR4	GGGTGAcagaAGGTCA	0.73±0.13
	+12575	DR4	ATGTCAagagAGTTCA	ND
	**+18783**	DR4	**AGGACAcacgAGGTCA**	**2.08±0.09**
*Klf9*	**−5206**	DR4	**GGTTCAtttgAGGACA**	**7.13±1.04**
	**−3763**	DR4	**AGGTGAagtgAGGTCA**	**6±0.67**
	−19085	IR0	CGGTCATGACCC	0.66±0.10
*Pfkfb3*	−23043	DR4	AGGCCAccctAGGTCA	ND
	−21724	DR4	AGTACAcaggAGTTCA	ND
	−13326	DR4	AGGTCAggagAATTCA	ND
	−1305	DR4	AGGCCAgccaGGGTCA	ND
	+519	DR4	AGGTCAaggtAGGTCT	ND
	+15186	DR4	GGTTAAggcaGGGTCA	ND
*Tgm2*	−10668	DR4	AGGTCAgcaaAAGTCA	0.82±0.16
	+5402	DR4	GGGTCTaaagGGGTCA	ND
	−13773	IR0	AGGTCATGACCT	ND
*Plp1*	−22862	DR4	GGTGCAgctgGGTTCA	ND
	−722	ER6	TGACCTtggcacAGGTCT	ND
	+5330	DR4	AGGGCAtttaAGTTCA	ND
*Plxna2*	+10679	ER6	TGACCAaaccttAGTTCA	ND
*Snap25*	Not found	N/A	N/A	N/A
*Spata13*	−13751	IR0	AGGTCAAGACCT*	ND
*Vnn1*	−1676	DR4	AGTTAAgaggAGGTCA	ND
	+23666	DR4	GGGCCAaataAGTTCA	ND
	+21041	DR4	AGGTCAgggtGGGTCA	ND
*Zbtb20*	−17282	DR4	GGTTCTtacaAGTTCA	ND
	−11201	DR4	GGTTCAcagaGGGCCA	ND
	−10524	IR0	AGTTGATGACCT	ND

* Identified using NUBISCAN. Bold characters correspond to TREs with more that 2-fold enrichment after C17.2/TRα1 cells ChAP. ND: not determined, N/A: not relevant. Mean ± SD for three independent experiments.

### In vivo T3 Target Genes Expression and Regulation by TRα1

**Figure 2:**
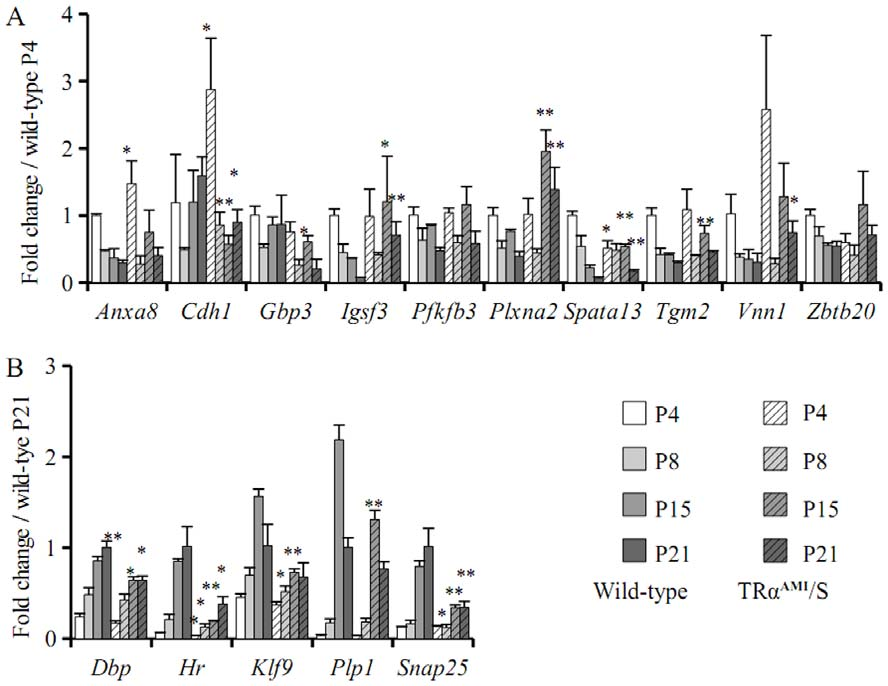
Kinetics of T3 target genes expression in wild-type and *TRα^AMI^/S* mice in the cerebellum as measured by Q-RT-PCR. Expression levels were calculated for each target gene by Q-RT-PCR in wild-type and *TRα^AMI^/S* littermates at P4, P8, P15 and P21 (minimum 3 animals of each genotype for each time point). Data are expressed as mean ± SD using wild-type P4 values (A), for genes with decreasing or stable expression levels over time or P21 (B), for genes with increasing expression levels, as a reference for each genotype. *p<0.05; **p<0.01 for comparisons between wild-type and *TRα^AMI^/S* mice for each time point (Student’s t-test).

As *Thra* is expressed in all cerebellum cell types, we used transgenic mice which express a dominant negative mutation of the TRα1 receptor (TRα1^L400R^) to address the *in vivo* relevance of the gene regulation observed in cultured cells. TRα1^L400R^ can be expressed from the so called *TRα^AMI^* allele of *Thra* only when a transcription stop cassette is deleted by *CRE/loxP* recombination. In *TRα^AMI^*/S mice, excision takes place soon after fertilization, and TRα1^L400R^ expression is nearly ubiquitous. *TRα^AMI^*/S mice display the defects in EGL migration and Purkinje cells arborization, which define congenital hypothyroidism in post-natal cerebellum [14], [16]. Q-RT-PCR of cerebellum RNA showed that significant alterations of gene expression between wild-type and *TRα^AMI^*/S mice at various developmental stages for the identified genes, indicating that these genes can be regulated by liganded TRα1 during cerebellum development, at least at one developmental stage (Figure 2). These results confirmed the predominant *in vivo* function of TRα1 and the relevance of the cell culture systems that we used. Expression kinetics revealed a variety of expression patterns, corresponding to the dynamic changes that occur during cerebellar development, and suggested that, for each gene, T3 sensitivity is restricted to a temporal window, sometimes limited to one time point. For some genes, opposite effects on expression of a target gene in wild-type and *TRα^AMI^*/S were observed at different time points, suggesting that a combination of different T3-mediated regulation mechanisms takes place during development. These variations in mRNA levels can however be influenced by modifications in cellular composition of the cerebellum due to the adverse developmental effects of TRα1^L400R^ and subsequent bias in whole cerebellum mRNA content.

To address this last possibility, we examined expression patterns for the identified target genes, using adult and fetal gene expression databases (Allen Mouse Brain Atlas, http://mouse.brain-map.org/welcome.do and Gensat, http://www.ncbi.nlm.nih.gov/projects/gensat/) and cell-type specific transcriptome analysis based on tagged ribosomes immunoprecipitation [32] (Table 3). Preferential *Anxa8, Plxna2* and *Spata13* expression seems to occur in granular cells and in their EGL precursors. Therefore, the observed increase in *Anxa8, Plxna2* and *Spata13* mRNA at P21 might at least in part reflect the persistence of EGL in *TRα^AMI^*/S cerebellum at late stage, rather than a transcriptional regulation. Although granular neurons and their precursors represent the majority of cells present in cerebellum and in the primary cell cultures that we initially used, it is striking that the number of genes found to be expressed preferentially in this lineage is very limited (*Anxa8, Hr, Plxna2*). By contrast, we picked up specific expression of glial cells (*Plp1*) and GABAergic neurons (*Dbp, Pfkfb3, Tgm2*), although these cell types were clearly under-represented in the primary cells culture system that we initially used.

**Table 3:** Database analysis of expression patterns and gene functions.

Gene	Adult		Newborn			Enrichment factors from TRAP study							Phenotype of knock-out mice	
	Allen Brain atlas		Allen Brain atlas		GENSAT	PC	GC	GI	SC/BC	BG	AS/BG	OL	Phenotype	Ref.
	Location	Enrichedin PCL	P4	P14	P7									
*Anxa8*	PCL, BG	No	N/A	N/A	EGL, IGL								N/A	N/A
*Cdh1*	IGL	N/A	ML, IGL	ML, WM	N/A				6,7				Early embryonic death	[33]
*Dbp*	PCL, ML,WM	Yes	PCL, ML, IGL	PCL, ML,IGL	PCL			3,2	3,0	2,7	2,4		Altered circadian clock	[34]
*Gbp3*	Ubi	No	N/A	N/A	N/A								N/A	N/A
*Hr*	IGL, ML	N/A	IGL	IGL, ML	N/A	2,2	2,0			2,3		2,2	Hair and skin defects	[35]
*Igsf3*	Ubi	No	N/A	N/A	N/A								N/A	N/A
*Klf9*	Ubi	Yes	N/A	N/A	N/A		2,6	3,0					Mild neurological and behavioral phenotype	[36]
*Pfkfb3*	PCL	Yes	N/A	N/A	N/A	2,3		4,1					Early embryonic death	[37]
*Plp1*	WM	N/A	WM, IGL	WM, IGL	N/A							5,6	Subtle myelination defects	[38]
*Plxna2*	IGL	N/A	ML, IGL	ML, IGL	EGL, IGL		5,4						Neuronal migration defects	[39]
*Snap25*	IGL, ML,PCL	No	N/A	N/A	Ubi		3,5					2,5	Defective neurotransmitter release, perinatal death	[40]
*Spata13*	Ubi	No	N/A	N/A	EGL								N/A	N/A
*Tgm2*	Ubi	No	N/A	N/A	N/A	3,0			2,4				Faster progression of Huntington’s disease	[41]
*Vnn1*	Ubi	Yes	N/A	N/A	N/A								No obvious brain phenotype	[42]
*Zbtb20*	IGL, ML,WM	N/A	IGL	IGL, ML,EGL, WM	IGL, WM								Defective glucose homeostasis perinatal death	[43]

Abbreviations: AS: Astrocytes, BC: Basket cells, BG: Bergmann glia, EGL: external granular layer, GI: Golgi interneurons, IGL: internal granular layer, ML: molecular layer, OL:Oligodendrocytes, PC: Purkinje cells, PCL: Purkinje cell layer, SC: Stellate cells, Ubi: ubiquitous, WM: White matter. N/A: not available. First location is the principal location. Data from Allen Brain Atlas, GENSAT and reference [32].

### Cell Autonomous and Indirect Regulations

As TRα1^L400R^ expression in transgenic mice depends on the Cre/loxP recombination system, we can address whether the *in vivo* regulation by TRα1 on T3 sensitive gene is a cell autonomous process, a feature that is an important indicator of a direct transcriptional control, by expressing TRα1^L400R^ in defined cell types. We crossed *TRα^AMI^* mice with transgenic mice expressing the CRE recombinase in restricted cell populations. The *TRα^AMI^/O* mice expresses a tamoxifen inducible version of the recombinase in the granular neurons of the posterior lobules of the post-natal cerebellum (Otx2CreER^T2^, [44]) and we therefore expected an inhibition of TRα1 function in the granular cell lineage in this model. *TRα^AMI^/P* mice express TRα1^L400R^ during fetal life in Purkinje cells and GABAergic interneurons progenitors (Ptf1a-Cre, [45]). *TRα^AMI^/C* mice express the mutation in the oligodendrocytes and their committed precursors (Cnp-Cre [46]). Tamoxifen treatment of *TRα^AMI^/O* at P1 followed by Q-RT-PCR analysis at P8 revealed a change in gene expression only for *Cdh1* and *Klf9* (Table 4), in keeping with the putative expression patterns established previously. *TRα^AMI^/C* mice displayed deregulation of *Igsf3*, a possible indication of regulation in the oligodendrocytes lineage. However, most changes in gene expression were observed in *TRα^AMI^/P* mice, where TRα1^L400R^ expression was restricted to GABAergic neurons. *Cdh1, Dbp, Pfkfb3* and *Tgm2* are thus possibly regulated by TRα1 in neuronal cell types that were under-represented in the initial culture conditions. Interestingly, some of the changes in gene expression found in *TRα^AMI^/P* mice are clearly not cell autonomous consequences of TR1^L400R^ expression in GABAergic neurons, but affect expression either in oligodendrocytes (*Plp1)* or granular neurons *(Plxna2)*. This might reflect an indirect response of this last cell type to diffusible factors secreted by GABAergic neurons under T3 stimulation.

**Table 4:** Cell autonomous effect of *in vivo* expression of a dominant negative TRα1 mutation.

Genes	*TRα^AMI^/S*P15	*TRα^AMI^/C*P15	*TRα^AMI^/P*P15	*TRα^AMI^/S*P8	*TRα^AMI^/O*P8	*TRα^AMI^/P*P8
N = (WT/mutants)	7/4	3/5	5/3	4/3	3/3	4/5
*Anxa8*	2.04±0.90	0.90±0.22	1.15±0.47	0.58±0.26	1.04±0.90	**0.59±0.25** *
*Cdh1*	**0.48±0.11** **	1.26±0.53	**0.45±0.11** *	1.76±0.39	**0.68±0.13** *	1.03±0.63
*Dbp*	0.75±0.04	ND	**0.71±0.13** **	0.88±0.17	0.87±0.09	1.30±0.29
*Gbp3*	**0.71±0.11** *	ND	0.64±0.06	0.50±0.16	0.65±0.24	0.67±0.39
*Hr*	**0.22±0.13** **	0.70±0.36	**0.80±0.16** *	**0.61±0.07** *	0.81±0.26	1.82±0.61
*Igsf3*	**3.34±1.87** *	**7.55±2.12** **	0.88±0.15	0.93±0.08	1.02±0.08	0.57±0.28
*Klf9*	**0.47±0.17** **	0.91±0.50	1.23±0.49	0.74±0.16	**0.67±0.08** *	1.06±0.43
*Pfkfb3*	1.37±0.32§	ND	**0.75±0.19** *	0.95±0.17	0.80±0.12	**0.20±0.11** **
*Plp1*	**0.60±0.10** **	1.80±0.61	0.80±0.17	1.03±0.32	0.52±0.15	**0.41±0.16** **
*Plxna2*	**2.56±0.42** **	1.20±0.61	0.84±0.17	0.86±0.11	0.74±0.19	**0.51±0.22** *
*Snap25*	**0.43±0.13** **	ND	0.94±0.22	0.76±0.07	0.73±0.07	0.94±0.58
*Spata13*	**2.39±0.15** **	ND	0.99±0.25	0.90±0.17	0.73±0.32	1.52±0.34§
*Tgm2*	**1.76±0.28** **	0.84±0.23	0.95±0.28	0.96±0.03	0.92±0.05	**0.43±0.11** *
*Vnn1*	3.64±1.42	ND	0.91±0.10	0.75±0.20	1.16±0.05	1.16±0.66
*Zbtb20*	2.10±0.89	ND	0.97±0.09	0.59±0.21	0.75±0.42	1.19±0.23

*TRα^AMI^/S* data are reported from figure 2 for comparisons. ND: Not determined. Values are indicated as mean ± SD. Significant changes (Student T-test) are indicated in bold:

** : p<0.01,

* : p<0.05.

## Discussion

By using a cell culture system aimed at maximizing T3 response of cerebellum cells, we were able to identify new T3 target genes, which are either up- or down-regulated during normal cerebellum development. Among the genes identified by our screen, only four were already reported as being regulated by T3, sometimes in other brain areas and at different stages: *Hr* [47], *Snap25* [48], *Dbp* [49] and *Klf9* [50]. Among the identified genes, some are regulated during early development, possess TREs in their regulatory sequences, which can be actually occupied by TRs, and are sensitive to the expression of a dominant negative TRα1 mutation in a cell-autonomous manner. These genes *(Hr, Dbp, Klf9, Gbp3)* are, beyond any reasonable doubt, direct TR targets. For the others the demonstration is only partial and alternative explanations for changes in genes expression are possible. In some cases (*Plp1*, *Plxna2*, *Tgm2*), slow kinetics could be an indication that they are secondary targets, activated by transcription factors which expression is itself regulated by T3, or by some of the neurotrophic signaling pathways that are activated by T3. Nevertheless, this study provides with a list of genes that may be important for cerebellar development and which role in this process would be worth investigating.

This study is at least the fourth attempt to identify T3 target genes in the cerebellum by broad transcriptional analysis, and there is little overlap between datasets. Although the discrepancies between the present and previous studies might in part be due to technical reasons, we believe that most differences can be explained by the fact that the repertoire of TRs target genes is highly dependent on cell types and, as indicated by our *in vivo* data, developmental stage. Here we favored the discovery of genes regulated by T3 in neurons, especially in the most abundant granular neurons, whereas previous attempts focused on whole cerebellum at early [18] or late [20]–[22] time points. Interestingly, only two of these genes (*Hr, Klf9*) have been identified as T3 target genes in neurons from several brain areas and at different stages in mice and even in frogs [22], [26], [50], [51], [52] and seem to possess the singular property to be inducible by T3 in various cellular contexts.

Current knowledge provides few indications that the gene regulations that we observed might significantly impact cerebellar development. However, *Klf9* knock-out mice show a deficit in the rotarod test, a sign of cerebellar disorder [36]. Its function in granular neurons remains unknown. *Zbtb20* encodes a transcription factor able to specify neuronal identity [53], [54]. *Plp1* is a gene encoding one of the major myelin component, proteolipid protein, expressed in oligodendrocytes. Thyroid hormone is known to be essential for oligodendrocytes differentiation [10] and thus for *Plp1* expression [55]. According to our data, *Plp1* is however unlikely to be a direct T3 target gene, and its *in vivo* down-regulation might just reflect a delay in oligodendrocytes differentiation. The fact that we detect changes in its expression in microarrays might be due to a less oligodendrocyte-restricted expression that previously known [56]. According to our observation *Plxna2* gene is also sensitive to T3 signaling *in vivo,* but its deregulation by expressing the mutant TRα1 only in GABAergic neurons although this gene is expressed in granular cells suggest that *Plxna2* is not a direct TRα1 target gene. It encodes a semaphorin receptor family member, which, by binding to its ligand, SEMA6A, promotes granule cells migration [39]. For both *Plp1* and *Plxna2*, some unknown primary event is triggered by T3, possibly in Purkinje neurons, as indicated by the *TRa^AMI^/CRE* models. Purkinje neurons cells secrete a number of neurotrophic and growth factors that coordinate cerebellum development [57]. The majority of the other newly identified TR target genes encode proteins with known functions, relevant to synaptic transmission (SNAP25), transcription factors (Zbtb20, DBP), adhesion and extracellular matrix proteins (ANXA8, CDH1, TGM2), signal transduction (Spata13) and cell survival (Tgm2). However, mouse genetics have not yet established their involvement in cerebellum post-natal development. The metabolic function of the *Pfkfb3* gene product, 6-phosphofructo-2-kinase/fructose-2,6-biphosphatase 3, is well known. This enzyme regulates the steady-state concentration of fructose-2,6-bisphosphate, a potent activator of a key regulatory enzyme of glycolysis, phosphofructokinase. Glycolysis is sensitive to T3 in several cell types, and a key parameter for neuronal activity. Glucose consumption was found to be reduced in the brain of mice expressing a TRα1 dominant-negative mutation [58]. Overexpression of *Pfkfb3* is however detrimental to neurons [59] as it favors the accumulation of toxic free radicals, a possible consequence of T3 excess in several systems [60]. This provides a possible explanation for increased neuronal cell death in hyperthyroid post-natal cerebellum [18]. As we found little glucose consumption in our primary cultures (data not shown), pyruvate being probably used as a substitute for energetic metabolism, we cannot address the metabolic consequence of *Pfkfb3* stimulation in our *in vitro* system.

Our initial broad survey identified a small number of genes in T3 sensitive genes in granular neurons, whereas it revealed T3 response of genes expressed in Purkinje neurons, and even in oligodendrocytes. This is a striking observation, considering that this two last cell types were a small minority of the cultured cell population and that EGL persistence at P21 and granular cells defects in differentiation and migration are the major landmarks of hypothyroidism. The fact that transcriptome analysis was sensitive enough to detect variations of rare mRNA prompts us to suggest that the total number of T3 target genes in granular neurons is very small, although it includes two of the best characterized examples: *Hr* and *Klf9*. That few genes are regulated by T3 in the granular cell precursors which populate the EGL is consistent with recent results showing that *Thra* expression is weak in the proliferating granular cells precursors [61], [62]. However, this explanation does not hold for post-mitotic granular neurons, but granular cells were mainly in precursor state when we did our primary cultures (P1–P4). The broad survey that we performed convinces us that most of the T3 direct response does not occur in the granular lineage that represents the vast majority of the cerebellum cell population at this stage, although thyroid hormone could have a major role on granular cell terminal differentiation at later stages. At early stages, our data suggests that T3-mediated gene regulation is mainly taking place in few cell types, including Purkinje cells, that play a pivotal role in a network of cellular interactions, mediated by direct contacts and neurotrophic factors secretion. The small number of target genes suggests that the cellular alterations observed in hypothyroid cerebellum are mainly indirect consequences of minor initial imbalance in gene expression, and that T3 controls the expression of few key developmental genes in cerebellum neurons. If our interpretation is correct, congenital hypothyroidism in cerebellum might be viewed mainly as desynchronization of the cellular interactions which govern proper neurodevelopment.

## Materials and Methods

### Animals

All animals used in this study were housed, raised, bred and used in accordance with European directive 86/609/EEC and in compliance with national and international rules and laws on animal welfare. Heterozygous mice expressing the TRα1^L400R^ ubiquitously were produced by using the sycp1CRE transgene expressed in spermatogonia [14]. Cell specific recombinations were obtained by crossing TRα^AMI^ mice with OtxCRE-ER^T2^ [44], Ptf1aCRE [45], CnpCRE [46]. Recombination patterns were verified by PCR [14] and by crossing with reporter transgenic ROSAYFP mice and immunocytochemistry [63].

### Primary Cultures of Newborn Mice Cerebellar Neurons

Newborn mice (P1–P4) were decapitated and the cerebellum was rapidly recovered in a Petri dish containing HBSS medium without Ca^2+^or Mg^2+^supplemented with 1 mM pyruvate and 10 mM Hepes. Cerebella were pooled and then washed and dissociated in 1 mL of the same medium. 2 mL of HBSS (with Ca^2+^and Mg^2+^)/1 mM pyruvate/10 mM Hepes was added and cells were centrifuged 1 min at 200 g. Supernatant was removed, cells were resuspended in Neurobasal medium containing 2% B27 supplement, 0.5 mM L-glutamine and 0.5% penicillin-streptomycin and plated in 24-wells plates coated with poly-lysine (0.05 mg/mL). All cell-culture media were purchased form Gibco-Invitrogen.

Cultures were maintained for 48 hours after seeding to favor neuronal survival at the expanse of glial cells and then treated with 0.1 µM T3 for 6, 16, 24 and 48 hours (one culture for each time point). Control cultures were submitted to the same scheme of medium change without addition of T3. Batches of cells were allowed to grow for up to 10 days and screened for expression of neuronal or glial markers by immunohistochemistry. This set of cultures and another set of primary cultures comprising the same conditions have been used for confirmation by Q-RT-PCR.

### RNA Extraction

Total RNA was extracted from cell cultures or mice cerebella with the Qiagen RNeasy kit according to manufacturers recommendations. RNA qualities and quantities were verified by capillary electrophoresis (BioAnalyser, Applied Biosystems) before reverse-transcription.

### Microarrays Hybridization

1 µg of each RNA was reverse transcribed and cDNA were submitted to one round of linear amplification before hybridization to whole-genome mice Affymetrix microarrays (GeneChip Mouse Genome 430 2.0 Array). One microarray was used for each condition. Data were analyzed using the Affymetrix GCOS software, data were normalized by Affymetrix MAS5.0 software, providing an estimation of expression levels for each probe-set and a p-value of the significance of the expression change. Data were pairwise compared for each time point between a T3-treated culture and a matched untreated culture prepared simultaneously from the same cell batch. This work was done by the Bio-informatics and Expression Analysis core facility at Karolinska Institutet (Sweden).

### Quantitative Reverse Transcription-PCR (Q-RT-PCR)

For Q-RT-PCR on mouse cerebella, 1 µg of each RNA sample was reverse-transcribed using MLV reverse-transcriptase (Promega). Quantitative PCR were then performed in 96 wells-plates using the *hypoxanthine guanine phosphoribosyl transferase* gene as a reference after carefully checking that our conditions did not significantly modify its expression level (less than one Ct of difference between compared samples, see also [22]) and the following mix: 4 µL of primers (see primers list in Table S1) mix 1.2 mM, 6 µL of SYBRGreen mix (either Qiagen Quanti-tect mix or Biorad iQ supermix) and 2 µL of cDNA diluted 1 in 20. A standard curve was made for each gene and each measure was made in triplicates. Melting curves were analyzed to ascertain homogeneity of the amplified products. Expression levels were calculated using the 2^−ΔΔ(Ct)^method [64]. Statistical differences have been calculated using unpaired Stutent t-test assuming different group sizes and variances.

### C17.2 Culture and C17.2/TRα1 Cells

C17.2 neural cells [18] were grown in DMEM medium (4.5 g/L glucose, glutamine, pyruvate) containing 10% FBS, 5% horse serum, 1% glutamine, 1% penicillin-streptomycin. Cells proliferate in this medium and were split 1/10 each week. Sera used for cell culture were deprived of T3 either by activated charcoal or resin treatment. When cultured without serum, serum removal was done simultaneously with T3 treatment. For testing target gene expression after transfection and in medium with or without serum, three independent experiments have been done.

pCEMM-GS-TRα1 was constructed by inserting the murine TRα1 reading frame amplified by PCR into the BamHI site of pCEMM-NTAP [65] create a reading frame encoding a GS-TRα1 protein, tagged at its N-terminus by a fragment of protein G. A downstream *IRES-gfp* cassette located on the transcription unit ensured co-expression of a green fluorescent protein. pCEMM-GS-TRα1 was co-transfected into C17.2 cells with pPGK-Puro and puromycine resistant cells were submitted to two rounds of sorting to select cells with stable expression of both gfp and GS-TRα1. This was further confirmed by Western blotting using an anti-TR antibody (Santa Cruz).

### Chromatin Affinity Purification (ChAP)

This method differs form chromatin immune-precipitation (ChIP) in that it does not involve an antibody directed against the DNA-binding protein of interest but relies on affinity binding between the tagged protein and other molecules bound to beads (in our case the protein G tag and IgG beads, see [65]). T3-treated or non-treated C17.2 cells were rinsed with PBS and cross-linked with 1% paraformaldehyde in PBS for 10 minutes at 37°C. Cells were then harvested, and stored at −80°C as pellets. Cell pellets were resuspended in 300 µL lysis buffer (1% SDS, 10 mM EDTA, 50 mM Tris pH 8) and sonicated 8 min (30s ON/30s OFF, high intensity) with a bath sonicator (Bioruptor, Diagenode). A small aliquot of cell lysate was analysed by gel electrophoresis and size of DNA fragments verified to be between 200 and 800 bp. 100 µL of the cell lysate was kept as input and the rest was diluted 1 in 10 in dilution buffer (1% Triton X-100, 1 mM EDTA, 150 mM Na Cl, 20 mM Tris pH 8) and incubated with IgG-coated magnetic beads (Invitrogen) at 4°C overnight. Negative control was obtained with non-transfected C17.2 cells. Beads were then rinsed twice in TSE1 buffer (0.1% SDS, 1% Triton X-100, 2 mM EDTA, 150 mM NaCl, 20 mM Tris pH 8), three times in TSE2 buffer (0.1% SDS, 1% Triton X-100, 2 mM EDTA, 500 mM NaCl, 20 mM Tris pH8), twice in LiCl wash buffer (0.25 M LiCl, 1% NP-40, 1% Sodium deoxycholate, 1mM EDTA, 1 mM Tris pH 8), twice with TE (10 mM Tris-HCl, 1 mM EDTA pH 8) and DNA fragments were eluted in 0.1 M NaHCO_3_, 1% SDS. Cross-link was reversed by overnight incubation at 65°C in 200 mM NaCl. DNA was purified using the Qiagen minElute kit, and quantified by Q-PCR as described above. Calibration curves were made with dilutions of input DNA and purification rates were calculated using the 2^−ΔΔ (Ct)^ method as input percentages. For each gene, a distal promoter DNA sequence was used as a control for non-specific background binding (see Table S2 for position of amplified sequence for both putative TREs and negative control). Enrichment was calculated as the ratio between input percentages of specifically bound quantity and non-specific binding. Preliminary experiments, using non-transfected cells, allow to set a threshold of 2-fold enrichment, as a minimum to ascertain genuine receptor binding (see Table S2 for primer sequences). Non-transfected cells gave no enrichment and no increase in purification in the precipitated fraction for any putative TRE (data not shown). Each putative has been tested on three independent ChAP experiments and results were well reproducible when enrichment was observed.

## Supporting Information

Table S1 **Q-RT-PCR primers used for gene expression experiments.**
(DOC)

Table S2 **Q-RT-PCR primers used for ChAP analysis.**
**(**DOC)
