# Supplementary material for: Genome-Wide Search Reveals the Existence of a Limited Number of Thyroid Hormone Receptor Alpha Target Genes in Cerebellar Neurons
Source: PLoS One. 2012 May 7;7(5):e30703. doi: 10.1371/journal.pone.0030703 (PMC3346809; doi:10.1371/journal.pone.0030703)
Supplement: Table S1 — Q-RT-PCR primers used for gene expression experiments. (DOC) [file pone.0030703.s001.doc]

Supplementary table 1: Q-RT-PCR primers used for gene expression experiments.

| **Gene** | **F primer** | **R primer** |
| --- | --- | --- |
| ***Anxa8*** | CCCTGGAAGAAGACATCCAA | CTGCGTGTGCACAGGATAGT |
| ***Cdh1*** | GCCGGAGAGGCACCTGGAGA | GGCGCGGACGAGGAAACTGG |
| ***Dbp*** | GAAGGCAAGGAAAGTCCAGGTGCC | GCTCCTGCCGCACAGCCA |
| ***Gbp4*** | TAGCACAGCCTCTGGTGGTGGT | GCCTTCAGTGTCCAAAAGGACCAGG |
| ***HPRT*** | CAGCGTCGTGATTAGCGATG | CGAGCAAGTCTTTCAGTCCTGTCC |
| ***Hr*** | GCCTTGCTTCCTATGATTGTCTCC | AGAGGTCCAAGGAGCATCAAGG |
| ***Igsf3*** | AACATCCCCATCGTCGTGCTGC | ACATGACATTGCTGCGGCGGT |
| ***Klf9*** | CACGCCTCCGAAAAGAGGCACAA | CTTTTCCCCAGTGTGGGTCCGGTA |
| ***Pfkfb3*** | CAGCTTTGAGGAGCGTGTGGCT | GGCACAAGGCAGGCTGTGGAAA |
| ***Plp1*** | GGCGACTACAAGACCACCATCTGC | ACACCAGGAGCCATACAACAGTCAGG |
| ***Plxna2*** | TGCAAATCAAGGAGCGGTTGCAGT | TACAGGGTCAGTCCCTCCACAGGA |
| ***Snap25*** | GGGCAGAGCTCACGTTGCATTGA | GGCCAGCAAGTCAGTGGTGCTT |
| ***Spata13*** | TTGGACACGCTGGCTTTGGAGC | ACCCCCATTCACGCCGGTAACT |
| ***Tgm2*** | TGCTGGGTGTTTGCAGCGGT | TGTTGCTCTCCAGCTCCCCGAA |
| ***Vnn1*** | CTCGCTCAGCGGCACTTTTGGA | GGATGCCCAGTCCTTCCCATACAAC |
| ***Zbtb20*** | CCAGCCCTCATCCACTCGACACAT | GCGAACCGTCACGTCACAGAAGT |

F : Forward orientation R : Reverse. All PCR products are less than 300 bp long.
