# Supplementary material for: Genome-Wide Search Reveals the Existence of a Limited Number of Thyroid Hormone Receptor Alpha Target Genes in Cerebellar Neurons
Source: PLoS One. 2012 May 7;7(5):e30703. doi: 10.1371/journal.pone.0030703 (PMC3346809; doi:10.1371/journal.pone.0030703)
Supplement: Table S2 — Q-RT-PCR primers used for ChAP analysis. (DOC) [file pone.0030703.s002.doc]

Supplementary table S2: Q-RT-PCR primers used for ChAP analysis.

| Gene | Chr. | TSS | Position TRE | Type TRE | Forward primer | Reverse primer |
| --- | --- | --- | --- | --- | --- | --- |
| Anxa8 | 14+ | 34899204 | -13935 | DR4 | CCACTGTGCCCCATTTTGCTAGTCTC | TCATGGACAGAACCTAGCCCAGGAC |
|  |  |  | -8825 | DR4 | TGGAACAGAGGTCCCTGATGGCA | AGCCAGACTGATGCGCTCCAAC |
|  |  |  | -7406 | DR4 | CCCTCCATCTTTCAGCACTGGAGC | GGGTGTCTGGGCCTGGTCAGATTTA |
|  |  |  | -314 | DR4 | AGACACAGTGCACCCCAGGGA | GCGCGCAGCCCTTCTGCTTA |
|  |  |  | -10383 | CTRL | AGGAGCATCAACAAGGCTGGCAC | AGCGAAGCCGGGATAGCCTCC |
| Cdh1 | 8+ | 109127264 | -9477 | DR4 | ACACCTTAGACTGGAAACCCT | TGATCCCAGCACTCAGGA |
|  |  |  | -2600 | DR4 | GTCACTACTGCACAGAGCCCCAAC | ACAGGTCAGGTCTAATGCACCCTTCA |
|  |  |  | +1784 | DR4 | GTAGGGAATGGCTGGGAGCAAGG | TCCCAGAAAGGGATTGTGTGTGACTTC |
|  |  |  | +14093 | DR4 | CCCCCTTCTCTTGCTAATCCTTTCGCT | AGCAAGCACTCAGGAGTCATCTTGACA |
|  |  |  | +19817 | DR4 | TGGCTCTGTCCATCCAGCCTTCA | TGGAACAGTCCAAGGTCACGGGAG |
|  |  |  | +22206 | DR4 | ACCCAGCACCTTACCTGCTACACA | ACGCCTGAGTCTTTGAGGCCAAC |
|  |  |  | +8445 | ER6 | AGGGGACCCGGAAATGTAGCCA | TGTGCTGGCCCCTCTTCCCAAA |
|  |  |  | +5010 | CTRL | TGCTGAAAGCCGCCTTGGACAG | TGGCCCACATGAGCCCTAGTCA |
| Igsf3 | 3+ | 101182236 | +8259 | DR4 | TGGCACCTTGGCCCTGGTCTTA | TCTCCATGGAAAGTGCTTCCTGGCTAA |
|  |  |  | +9904 | DR4 | GCCTGGCTCACCTTTCCACTGC | AAGGGGCTCCTCCGGTGGTC |
|  |  |  | +12575 | DR4 | GGATGGATAGGGCTTGGCATTCTTTGG | CACTGGTCACATTCATGGGGCATCC |
|  |  |  | +18783 | DR4 | CGTGTTGTGCTAAGAGGCGTGAGC | TTCAGGCTTCGTGGGAGATGAGCC |
|  |  |  | +7221 | CTRL | TGGAGCAGACGGTCGGTATGGA | ATAGAACTCGGGCTCAGGGGCA |
| Dbp | 7+ | 52960602 | -17879 | DR4 | ACAAAGCCACGCTGTATGACCTGC | ACGTAACTGAGCAGAGGAGCCGA |
|  |  |  | -10039 | DR4 | TCTGTGAGCACGCTGATGGGAAGA | GGTGGTTCCCACAACCCCTGTGAA |
|  |  |  | +18318 | DR4 | TTGACCACCAGCATCTGCCTGC | AACTCCCACCCTCCAGTGGCAT |
|  |  |  | -341 | DR4 / ER6 | AGCACGCGCAAAGCCATGTG | GCGTGAGGGGCTGAACTTGTGTA |
|  |  |  | -13793 -13659 | CTRL | ACGGGAGGGATGTGCAACCAGA | TAGGACTCACGTTGGCAGGGCA |
| Gbp3 | 3+ | 142223016 | +406 | DR4 / ER6 | TCTGCAAGATGTGGCGGGGTGA | GTGGCAAAGTAACAGCCGCCCAA |
|  |  |  | +10643 | IR0 | CCTCTGACTGATGCTCAGGGAGGTC | AGCCGGGCCAGCATCATTGT |
|  |  |  | +3185 | CTRL | ACCTGGTGGGAAAGGCAGCACA | GCAAGGCCAAGTGGAGGTCTTCAG |
| Klf9 | 19+ | 23215716 | -5206 | DR4 | TGCACGAGTTTGGGGCGGATTC | TGGGCCTGGCATCGCCCTTTTA |
|  |  |  | -3763 | DR4 | TGACATTCTACAGGCCCACCCTGG | ACCTCACTTCACCTCTCCCAGCCA |
|  |  |  | -19085 | IR0 | ACGAGCTGTGCTGGTGTGAGTC | TGCAGCTGAGGTTAGAGGTGGGTC |
|  |  |  | -14273 | CTRL | CACGGGAAAGGCTGGGTTGTGA | TTACTGTCTCTACCTCTGGGCCTGC |
| Tgm2 | 2- | 157972164 | -10668 | DR4 | ACCAGGCCCAACCTGGCTGTAA | GTCCACCATGCCAGCCTAAGCAA |
|  |  |  | -5677 | CTRL | AGAACCAGCCTCCACTGCCCAA | TGCCCAAAGGAACCCCTCGTCA |
